# Nicotine pouch use and associated characteristics among U.S. adults: Tobacco use supplement to the current population survey, 2022–2023

**DOI:** 10.1371/journal.pone.0362052

**Published:** 2026-11-11

**Authors:** Nicole A. Tashakkori, Nicole F. Pelletier, Joanne T. Chang, Carol H. Christensen, Rachel Grana Mayne, Margaret Mayer Sutherland, Karen A. Cullen

**Affiliations:** 1 Division of Population Health Science, Office of Science, Center for Tobacco Products, Food and Drug Administration, Silver Spring, Maryland, United States of America; 2 Tobacco Control Research Branch, Behavioral Research Program, Division of Cancer Control and Population Sciences, Rockville, Maryland, United States of America; Ohio State University, UNITED STATES OF AMERICA

## Abstract

**Introduction:**

Despite a recent rise in nicotine pouch sales, nationally representative data on demographic characteristics and patterns of use are lacking. We utilized the 2022–2023 Tobacco Use Supplement to the Current Population Survey to estimate the prevalence of nicotine pouch use among U.S. young adults and adults stratified by nicotine pouch use, demographic characteristics, and other tobacco product use patterns.

**Methods:**

Data were collected in September 2022, January 2023, and May 2023. We calculated weighted prevalence estimates and 95% confidence intervals of nicotine pouch use. Estimates were compared by patterns of use, age, other tobacco product use, and administration month.

**Results:**

Among respondents (N = 114,206), 2.8% (n = 3,542) reported ever nicotine pouch use and 0.4% (n = 539) reported current use. People who ever and currently use nicotine pouches were more likely to be male, non-Hispanic White, aged 35–54 years, and living in a metropolitan area. Current nicotine pouch use was most prevalent among people who currently use smokeless tobacco (10.5%), formerly used smokeless tobacco (3.6%), and who use any tobacco products (1.2%). Among those who reported ever-use of nicotine pouches the proportion reporting current use across successive cross-sectional study waves was 12.6% (September 2022), 15.4% (January 2023), and 19.7% (May 2023) and almost all people who use nicotine pouches reported other tobacco product use.

**Conclusions:**

Nicotine pouch use was low among U.S. young adults (18–24 years) and adults (≥25 years) in 2022–2023. Given the higher prevalence estimates of current nicotine pouch use among people who ever use in May 2023 than in September 2022, ongoing surveillance is important to monitor patterns of use.

## Introduction

Nicotine pouches are small, flavored pouches containing nicotine, but not tobacco leaf. These products, which are designed to be used orally, have increased in use over the past several years [1]. An increase in nicotine pouch sales was first seen in 2016, and sales have continued to rise since [2,3]. Despite this, little is known regarding patterns of use for this emerging product and the characteristics of adults who use them. Published literature from nationally representative data on adults who use nicotine pouches is limited, particularly related to in depth patterns and characteristics of use, such as brand.

Among youth, 2024 National Youth Tobacco Survey (NYTS) data show 1.8% of middle and high school students reported current nicotine pouch use, including 2.4% of high school and 1.0% of middle school students [4]. While some changes have been seen in the prevalence of use in recent years, overall, the prevalence of youth nicotine pouch use appears low [5,6]. Data from convenience samples (2021–2022) using online research panels show a higher prevalence of nicotine pouch use among adolescents and young adults aged 15–24, with estimates as high as 16% for ever use and 12% for current use [7,8]. Few population-based estimates have been published examining the prevalence of nicotine pouch use in the U.S. adult population and among people who currently use tobacco. According to studies examining 2022–2023 Tobacco Use Supplement to the Current Population Survey (TUS-CPS) data, the prevalence of ever nicotine pouch use was less than 3% and current nicotine pouch use was approximately 0.4% [9–11].

Among adults who use combustible tobacco, data from the September 2022 TUS-CPS found that 10.8% of people who currently smoke cigarettes ever used nicotine pouches and 1.0% currently used nicotine pouches [9]. Among people who formerly smoked, 6.7% reported ever use and 0.7% reported current nicotine pouch use [9]. Further, results from a recently published study by Delnevo and colleagues examining the 2022–2023 TUS-CPS found current some day smoking associated with increased odds of current and daily nicotine pouch use [11]. In 2021, results from a probability-based web panel found that 5.6% of adults who smoke ever used nicotine pouches and 16.8% reported interest in using nicotine pouches over the next six months [12]. Further, one study examining nicotine pouch use over two years in a sample of 15–24-year-olds in the U.S. noted that 73% of people who currently use nicotine pouches and 33% of those who ever, but not currently, used nicotine pouches reported current cigarette use [8].

Given recent increases in nicotine pouch sales and inconsistent use estimates among adolescents, young adults, and adults, it is important to further understand user characteristics and patterns of use associated with these products. As such, this study utilized 2022–2023 TUS-CPS data to estimate the prevalence of nicotine pouch use among adults (≥25 years) and young adults (18–24 years) stratified by demographic characteristics and other tobacco product use. While several papers have been published on nicotine pouch use using TUS-CPS data, this analysis builds on prior observations of the data through its in-depth assessment of differences among age groups in patterns of other tobacco product use and its detailed characteristic breakdown. This paper is the first nationally representative study to include estimates on the brand of nicotine pouch used. Results from this manuscript contribute to a greater understanding of these novel oral tobacco products and inform future surveillance and effective regulatory efforts.

## Methods

### Data source

This study utilized the 2022–2023 TUS-CPS [13]. The 2022–2023 TUS-CPS was the first survey wave to include questions about nicotine pouch use. Administered by the U.S. Census Bureau as part of the Current Population Survey, TUS-CPS is a nationally representative cross-sectional survey that uses stratified, multistage sampling procedures to collect data on tobacco use behaviors, attitudes, and policies from civilian, non-institutionalized persons aged ≥18. Each wave is collected across three nonconsecutive months (September 2022, January 2023, May 2023). Each month's data is weighted to the U.S. civilian, non-institutionalized population and can be analyzed independently or together with all three months. In full, TUS-CPS is the largest national survey of adult tobacco use. About two-thirds of respondents complete the questionnaire by telephone. Responses for the remaining third are obtained through in-person interviews. Proxy responses were collected for unavailable respondents. Due to the abbreviated nature of proxy responses, only self-respondents were included in this study sample. The study sample included all self-respondents and did not exclude indeterminate responses, including no response, don’t know, and refused.

### Measures

Ever nicotine pouch use was determined by asking, “The next question is about nicotine pouches. These are different from other smokeless tobacco products because they do not contain any tobacco leaf. These small white pouches contain nicotine which users place in their mouth. Some popular brand names include ZYN (pronounced “zin”), Rogue, on!, or Velo (pronounced “vee-low”). Have you ever used a nicotine pouch even one time?” Respondents who answered “yes” were asked: “Do you now use nicotine pouches every day, some days, or not at all?” Those who responded “every day” or “some days” were categorized as people who currently use nicotine pouches. Respondents who used nicotine pouches every day or some days (current use), were also asked: “On how many of the past 30 days did you use nicotine pouches?” Respondents could provide any full integer 0–30. If they reported use on ≥1 day(s) in the past-30-days (P30D), they were asked about brand and flavor of nicotine pouch used in the P30D.

For brand used most often in the P30D, respondents were asked: “During the past 30 days, what brand of nicotine pouches did you use the most often?” Only one choice was allowed for selection, including ZYN, on!, Velo, Rogue, Rush, NIIN, FRĒ, Bidi Pouches, or other. Due to low response rate, Rush, NIIN, FRĒ, and Bidi Pouches were top coded by the U.S. Census Bureau. Top coding assigns a masking value to those reporting an answer that is uncommon to protect confidentiality due to low reporting.

For nicotine pouch flavor(s), respondents who reported any P30D nicotine pouch use were asked, “During the past 30 days, when you used nicotine pouches, did you use [menthol flavor; mint flavor; clove, spice, or herb flavor; fruit flavor; alcoholic drinks (such as wine or other cocktails) flavor; candy, dessert, or other sweets flavor; tobacco flavor; unflavored type; some other type of flavor?].” Each of the nine flavor categories were assessed as separate yes/no questions, thus, respondents could report using multiple flavor categories. Due to low response rates, alcoholic drinks; candy, dessert, or other sweets flavor; tobacco flavor; and some other type of flavor were top coded to protect the confidentiality of those who reported using these flavors [13]. Top coded responses are represented collectively under the “top coded” category in tables.

Sociodemographic characteristics assessed include sex (male, female); race and ethnicity (non-Hispanic White, non-Hispanic Black, Hispanic, non-Hispanic other [Non-Hispanic American Indian and Alaskan Native, Asian, Hawaiian or Pacific Islander, and persons of multiple races are combined into one category (non-Hispanic other) due to sample size and analytic concerns]); age group (18–24, 25–34, 35–54, ≥ 55); educational attainment (less than high school, high school degree or GED, some college/associate degree, bachelor’s degree or above); U.S. region (Northeast, Midwest, South, West); and metropolitan area (metropolitan, non-metropolitan, not identified).

Nicotine pouch use was also assessed by other tobacco product use, such as current, former, and never cigarette, smokeless tobacco, and electronic nicotine delivery systems (ENDS) use; never use of any tobacco product besides nicotine pouches; and use of any tobacco product besides nicotine pouches. Current, former, and never cigarette use was determined by respondents reporting ever having smoked 100 lifetime cigarettes and whether they currently smoked cigarettes every day or some days. Current and former smokeless tobacco use was defined by people who ever use smokeless tobacco’s response to whether they currently used smokeless tobacco every day or some days. The 2022–2023 TUS-CPS survey defines smokeless tobacco as products that contain tobacco leaf and are used in the mouth. This includes moist snuff, dip, spit, chew tobacco, or snus. Current, former, and never ENDS use was determined by respondents reporting ever ENDS use and whether they currently used ENDS. TUS-CPS describes ENDS as battery-powered devices that usually contain liquid nicotine and produce vapor instead of smoke, encompassing disposable e-cigarettes, pod- or cartridge-based devices, tank-based e-cigarettes, and mod systems. People who never used any other tobacco products were considered to have never used any tobacco products besides nicotine pouches. People who used any tobacco products were those who currently used at least one tobacco product every day or some days, which could include nicotine pouches.

### Statistical analysis

Among people who currently use nicotine pouches, sociodemographic and patterns of product use were compared between some day and every day current use along with between young adults 18–24 years and adults ≥25 years. Among respondents who reported current nicotine pouch use, the distribution of sociodemographic and patterns of product use were analyzed by current, former, and never cigarette use; current and former smokeless tobacco use; never use of any tobacco product; and any tobacco product use. Additionally, we calculated the prevalence of nicotine pouch use by each month of survey administration (September 2022, January 2023, May 2023) to monitor changes in prevalence. Sociodemographic and product use characteristics among respondents who reported ever, current, and P30D nicotine pouch use were presented by survey administration month.

Unweighted sample sizes are presented alongside weighted prevalence estimates and 95% confidence intervals (CIs). Self-response weights were used to account for the probability of self-response and complex sampling design, while self-response replicate weights were used to estimate robust variances. For variance estimation, the analysis incorporated balanced repeated replication with Fay’s adjustment value of 0.5. Estimates were flagged if relative standard error (RSE) was ≥ 30% and suppressed if cell sample size was one or two for data confidentiality. Chi-square tests of proportions were used to compare characteristics between groups (every day vs. some day use of nicotine pouches; older adults vs. young adults; current vs. former cigarette smoking; current vs. former vs. never cigarette smoking; current vs. former smokeless tobacco use; September 2022 vs. January 2023 vs. May 2023 survey administration months). Data analyses were conducted using SAS version 9.4 callable SUDAAN v11.0.3.

## Results

Among all 2022–2023 TUS-CPS self-respondents (N = 114,206), 2.8% (n = 3,542, 95% CI: 2.7%, 3.0%) reported having ever used nicotine pouches, with 0.4% (n = 539, 95% CI: 0.4%, 0.5%) reporting current nicotine pouch use (Table 1). The gender distribution of respondents in the 2022–2023 TUS-CPS was approximately equal, with females comprising 51.24% and males comprising 48.76% of the overall sample [14].

**Table 1:** Nicotine pouch use among adults overall, and by demographic characteristics, brand, and flavor, 2022-2023 TUS-CPS, (N = 114,206).

	Ever users of nicotine pouches		Current users of nicotine pouches***		Every day users of nicotine pouches		Some day users of nicotine pouches		P-value^1^
	Unweighted no.	% (95% CI)	Unweighted no.	% (95% CI)	Unweighted no.	% (95% CI)	Unweighted no.	% (95% CI)	
**Prevalence of use in overall sample**	3,542	2.8 (2.7, 3.0)	539	0.4 (0.4, 0.5)**	256	0.2 (0.2, 0.2)	283	0.3 (0.2, 0.3)	
***Sociodemographic characteristics***									
**Sex**									0.25
Male	2,641	77.4 (75.7, 79.0)	470	89.2 (85.4, 92.1)	230	91.3 (85.7, 94.8)	240	87.7 (82.3, 91.5)	
Female	901	22.6 (21.0, 24.3)	69	10.8 (7.9, 14.6)	26	8.7 (5.2, 14.3)	43	12.3 (8.5, 17.7)	
**Race/Ethnicity**									0.01^†^
Non-Hispanic White	3,033	81.9 (80.0, 83.6)	485	85.7 (80.7, 89.6)	237	90.4 (83.4, 94.7)	248	82.1 (74.7, 87.7)	
Non-Hispanic Black	139	5.1 (4.2, 6.3)	14	4.6 (2.4, 8.4)*	^	^	13	8.0 (4.3, 14.4)*	
Hispanic	202	7.7 (6.5, 9.1)	26	7.2 (4.7, 11.0)	12	7.5 (3.8, 14.2)*	14	7.0 (4.0, 12.1)	
Non-Hispanic other^a^	168	5.3 (4.4, 6.3)	14	2.5 (1.3, 4.7)*	6	2.0 (0.7, 5.5)*	8	2.8 (1.3, 6.3)*	
**Age group (year)**									0.31
18-24	222	11.0 (9.6, 12.5)	62	19.4 (14.5, 25.6)	26	14.4 (9.2, 22.0)	36	23.2 (16.4, 31.6)	
25-34	789	23.4 (21.7, 25.2)	161	28.5 (24.0, 33.5)	77	30.0 (23.3, 37.8)	84	27.4 (21.3, 34.4)	
35-54	1,355	37.9 (35.9, 40.0)	219	36.5 (31.9, 41.3)	105	39.3 (32.5, 46.6)	114	34.4 (28.1, 41.2)	
≥55	1,176	27.7 (26.2, 29.3)	97	15.6 (12.7, 19.0)	48	16.2 (11.8, 21.8)	49	15.1 (11.3, 19.9)	
**Education**									0.20
<High school	209	5.4 (4.6, 6.4)	28	5.7 (3.5, 9.0)	10	3.8 (1.6, 8.5)*	18	7.1 (4.0, 12.1)	
High school degree or GED	1,008	32.4 (30.3, 34.5)	157	32.7 (27.3, 38.6)	83	39.8 (31.7, 48.5)	74	27.4 (20.0, 36.1)	
Some college/associate degree	1,254	33.9 (32.0, 35.9)	178	32.9 (27.5, 38.8)	87	30.6 (24.3, 37.7)	91	34.7 (26.5, 43.9)	
≥Bachelor's degree	1,071	28.3 (26.5, 30.1)	176	28.7 (24.0, 33.9)	76	25.8 (19.8, 32.8)	100	30.9 (24.4, 38.3)	
**Region**									0.26
Northeast	411	13.5 (12.1, 15.1)	56	14.6 (11.2, 18.9)	27	16.2 (10.7, 23.8)	29	13.4 (8.8, 20.0)	
Midwest	857	25.6 (23.7, 27.6)	137	27.6 (23.4, 32.3)	63	27.9 (21.4, 35.6)	74	27.4 (21.6, 34.0)	
South	1,116	34.3 (32.2, 36.5)	128	27.1 (22.2, 32.7)	50	21.8 (15.7, 29.6)	78	31.1 (24.5, 38.7)	
West	1,158	26.5 (24.7, 28.5)	218	30.6 (25.3, 36.5)	116	34.0 (27.0, 41.8)	102	28.0 (21.1, 36.3)	
**Metropolitan Area**									0.62
Metropolitan	2,629	80.9 (78.9, 82.8)	397	80.0 (74.6, 84.4)	179	77.6 (70.4, 83.4)	218	81.8 (73.9, 87.7)	
Non-metropolitan	868	17.9 (16.0, 20.0)	139	19.4 (15.0, 24.7)	76	21.9 (16.2, 28.9)	63	17.5 (11.8, 25.3)	
Not identified	45	1.2 (0.7, 2.0)	3	0.6 (0.1, 2.9)*	^	^	^	^	
***Tobacco use behaviors***									
**Cigarette smokers**									<0.001^†^
Current cigarette smokers	1,117	31.3 (29.5, 33.1)	119	21.6 (17.9, 25.8)	34	11.1 (7.3, 16.6)	85	29.5 (23.6, 36.3)	
Former cigarette smokers	1,614	43.9 (42.1, 45.7)	212	34.7 (30.4, 39.2)	134	51.3 (43.2, 59.3)	78	22.1 (16.9, 28.2)	
Never cigarette smokers	810	24.8 (23.0, 26.8)	208	43.7 (39.2, 48.4)	88	37.6 (30.2, 45.7)	120	48.4 (41.5, 55.4)	
Indeterminate	1	0.0 (0.0, 0.3)	0	0.0 (0.0, 0.0)	0	0.0 (0.0, 0.0)	0	0.0 (0.0, 0.0)	
**ENDS users**									0.03^†^
Current ENDS users	562	18.1 (16.6, 19.8)	89	20.7 (15.6, 26.8)	29	12.4 (7.5, 19.8)	60	26.9 (19.4, 36.1)	
Former ENDS users	1,326	39.7 (37.6, 41.8)	190	35.2 (30.2, 40.6)	99	38.5 (31.0, 46.7)	91	32.7 (25.6, 40.7)	
Never ENDS users	1,654	42.2 (40.2, 44.2)	260	44.1 (38.8, 49.5)	128	49.0 (41.1, 57.0)	132	40.4 (33.2, 48.0)	
Indeterminate	0	0.0 (0.0, 0.0)	0	0.0 (0.0, 0.0)	0	0.0 (0.0, 0.0)	0	0.0 (0.0, 0.0)	
**Smokeless tobacco users**									0.003^†^
Current smokeless tobacco users	445	12.3 (11.1, 13.6)	141	27.5 (22.9, 32.5)	45	18.0 (12.9, 24.7)	96	34.6 (28.0, 42.0)	
Former smokeless tobacco users	1,624	46.4 (44.3, 48.4)	227	40.1 (34.3, 46.1)	126	45.0 (36.7, 53.5)	101	36.3 (29.3, 44.0)	
Never smokeless tobacco users	1,468	41.2 (39.3, 43.2)	171	32.5 (27.3, 38.1)	85	37.0 (29.4, 45.3)	86	29.0 (22.7, 36.3)	
Indeterminate	5	0.1 (0.0, 0.2)	0	0.0 (0.0, 0.0)	0	0.0 (0.0, 0.0)	0	0.0 (0.0, 0.0)	
**Use in the past 30 days** ^**b**^	501	14.6 (13.0, 16.3)	501	92.2 (88.8, 94.7)	256	100.0 (100.0, 100.0)	245	86.3 (80.8, 90.5)	<0.001^†^
**Most often used brand in past 30 days**^**c**^ (only 1 selection allowed)									
ZYN			241	46.9 (40.7, 53.2)	123	43.0 (35.4, 50.9)	118	50.3 (41.7, 58.9)	0.19
on!			99	19.7 (15.7, 24.4)	51	23.5 (17.6, 30.5)	48	16.3 (11.1, 23.5)	0.14
Velo			19	5.1 (3.0, 8.5)	12	6.2 (3.0, 12.6)*	7	4.1 (1.9, 8.6)*	0.46
Rogue			48	8.8 (6.3, 12.1)	28	10.5 (7.1, 15.3)	20	7.2 (4.1, 12.5)	0.26
Other			80	16.5 (12.5, 21.4)	37	14.8 (9.4, 22.6)	43	17.9 (12.5, 24.9)	0.50
Missing/unknown			14	3.1 (1.8, 5.3)	5	2.0 (0.9, 4.3)*	9	4.1 (2.0, 7.9)*	0.20
**Flavor(s) used in past 30 days**^**c**^ (can select >1 flavors)									
Menthol			33	7.7 (5.2, 11.4)	17	8.1 (4.9, 13.3)	16	7.4 (3.9, 13.4)*	0.81
Mint			250	49.3 (43.3, 55.3)	126	45.6 (37.4, 54.0)	124	52.6 (44.6, 60.4)	0.22
Clove, spice, or herb			25	3.9 (2.1, 7.1)*	13	4.5 (2.2, 9.2)*	12	3.3 (1.4, 7.4)*	0.51
Fruit			46	6.8 (4.6, 9.9)	27	7.9 (4.7, 13.0)	19	5.8 (3.3, 10.1)	0.45
Unflavored			69	18.3 (14.0, 23.5)	31	13.5 (8.5, 20.7)	38	22.5 (16.0, 30.6)	0.07
Top coded			119	22.4 (17.5, 28.3)	67	27.7 (21.4, 35.1)	52	17.7 (11.4, 26.5)	0.04^†^

Note: unweighted number and weighted % (95% CI) presented.

The sample (N = 114,206) includes all self-respondents with no exclusions made for indeterminate responses, including no response, don’t know, and refused.

**Every day plus some day users. Current some day or every day use is determined among respondents by the question “Do you NOW use nicotine pouches every day, some days or not at all?”

**The individual weighted prevalence estimates for every day and some days are rounded independently and may not sum to the total shown.

*Relative standard error (RSE) ≥30%.

^Cells with a sample size of 1 or 2 are suppressed.

^1^P-value for comparison between every day and some day users of nicotine pouches.

†Indicates statistical significance (p < 0.05) from the χ2 test.

^a^Non-Hispanic American Indian and Alaskan Native, Asian, Hawaiian or Pacific Islander, and persons of multiple races are combined into one category due to sample size and analytic concerns.

^b^Past 30-day use is determined among respondents by the question “On how many of the past 30 days did you use nicotine pouches?” If a respondent used nicotine pouches on  ≥1 of the past 30 days, they were characterized as having nicotine pouch use in the past 30 days.

^c^Data on brand and flavors used were collected among every day users or some day users who used in past 30 days.

Due to a low response rate, Rush, NIIN, FRĒ, and Bidi Pouches were top coded.

Due to a low response rate, alcoholic drinks; candy, dessert, or other sweets flavor; tobacco flavor; and some other type of flavor were top coded and are collectively represented in the top coded category.

Respondents who reported ever nicotine pouch use were predominantly male (77.4% [95% CI: 75.7%, 79.0%]), non-Hispanic White (81.9% [95% CI: 80.0%, 83.6%]), and aged 35–54 (37.9% [95% CI: 35.9%, 40.0%]), and reported former cigarette (43.9% [95% CI: 42.1%, 45.7%]), never ENDS (42.2% [95% CI: 40.2%, 44.2%]), and former smokeless tobacco use (46.4% [95% CI: 44.3%, 48.4%]) (Table 1).

Similarly, respondents who reported current use of nicotine pouches were predominantly male (89.2% [95% CI: 85.4%, 92.1%]), non-Hispanic White (85.7% [95% CI: 80.7%, 89.6%]), aged 35–54 (36.5% [95% CI: 31.9%, 41.3%]), and reported never ENDS (44.1% [95% CI: 38.8%, 49.5%]) and former smokeless tobacco use (40.1% [95% CI: 34.3%, 46.1%]). People who currently use nicotine pouches are more likely to be male than people who ever use nicotine pouches (89.2% vs. 77.4%). Further, we observed a higher proportion of people who never smoke cigarettes among people who currently use nicotine pouches (43.7% [95% CI: 39.2%, 48.4%) compared to people who ever use (24.8% [95% CI: 23.0%, 26.8%) (Table 1).

Among respondents who reported current nicotine pouch use in the P30D, the most common brand used was ZYN (46.9% [95% CI: 40.7%, 53.2%]), followed by on! (19.7% [95% CI: 15.7%, 24.4%]). The most common nicotine pouch flavor used was mint (49.3% [95% CI: 43.3%, 55.3%]), followed by unflavored (18.3% [95% CI: 14.0%, 23.5%]).

Respondents who reported current use were further stratified by every day and some day nicotine pouch use. Both respondents who reported every day and some day use were more often male, non-Hispanic White, aged 35–54, resided in a metropolitan area, and reported never ENDS and former smokeless tobacco use (Table 1). However, respondents who reported every day use more often had a high school degree or GED (39.8% [95% CI: 31.7%, 48.5%]), while people who use on some days predominately had some college/associate degree (34.7% [95% CI: 26.5%, 43.9%]). Respondents who reported every day use more often reported living in the West (34.0% [95% CI: 27.0%, 41.8%]), while people who use on some days more often lived in the South (31.1% [95% CI: 24.5%, 38.7%]). Moreover, respondents who reported every day nicotine pouch use more often reported former cigarette smoking (51.3% [95%CI: 43.2%, 59.3%]) and people who used on some days more often reported never smoking (48.4% [95% CI: 41.5%, 55.4%]). As observed among people who currently use overall, the most common brand among respondents who reported every day or some day use and those who also reported use in the P30D was ZYN [every day: (43.0% [95% CI: 35.4%, 50.9%]); some day: (50.3% [95% CI: 41.7%, 58.9%])]. Among people who reported every day use, the second most common brand used was on! (23.5% [95% CI: 17.6%, 30.5%]). However, among people who reported some day use, “other” was the second most reported brand (17.9% [95% CI: 12.5%, 24.9%]), followed by on! (16.3% [95% CI: 11.1%, 23.5%]). The most common flavor used by people who use every day and some day who also reported P30D use was mint [every day: (45.6% [95% CI: 37.4%, 54.0%]); some day: (52.6% [95% CI: 44.6%, 60.4%])], followed by unflavored [every day: (13.5% [95% CI:8.5%, 20.7%]); some day: (22.5% [95% CI: 16.0%, 30.6%])] (Table 1).

When the prevalence of current nicotine pouch use was stratified by age group (young adults aged 18–24 years vs. adults aged ≥25 years), young adults (aged 18–24) more often reported current use of nicotine pouches (0.7% [95% CI: 0.5, 1.0]) than adults (aged ≥25) reporting current use (0.4% [95% CI: 0.4, 0.5]). Young adults (aged 18–24) more often reported never smoking cigarettes (75.0% [95% CI: 61.3%, 85.0%]) and adults (aged ≥25) more often reported former cigarette smoking (39.9% [95% CI: 34.5%, 45.5%]). Young adults (aged 18–24) more often reported current ENDS use (43.9% [95% CI: 26.9%, 62.4%]) and adults (aged ≥25 years) more often reported never ENDS use (48.9% [95% CI: 43.0%, 54.7%)]). Use of the brand ZYN was reported among a significantly higher proportion of young adults (aged 18–24) (65.3% [95% CI: 49.0%, 78.7%]) than among adults (aged ≥25 years) (42.7% [95% CI: 37.0%, 48.7%]) who reported P30D nicotine pouch use. Use of the brand on! was reported among a higher proportion of adults (≥25 years) (21.0% [95% CI: 16.8%, 26.1%]) compared to young adults (aged 18–24 years) (13.6% [95% CI: 5.7%, 29.2%]). Additionally, there was a significantly higher proportion of “other” brand use among adults (≥25 years) (18.2% [95% CI: 14.0%, 23.4%]) compared to “other” brand use in young adults (18–24 years) (8.6% [95% CI: 3.1%, 21.9%]). There was a significantly higher proportion of mint flavor use among people who used in the P30D in young adults (68.9% [95% CI: 51.8%, 82.1%]) compared to adults (44.8% [95% CI: 38.6%, 51.2%]) (Table 2).

**Table 2:** Current nicotine pouch use** overall and by age group (18-24 and ≥25 Years), 2022-2023 TUS-CPS.

	Overall		Young adults aged 18–24 years		Adults aged ≥25 Years		P-value^1^
	Unweighted no.	% (95% CI)	Unweighted no.	% (95% CI)	Unweighted no.	% (95% CI)	
**Prevalence of use in overall sample**	539	0.4 (0.4, 0.5)	62	0.7 (0.5, 1.0)	477	0.4 (0.4, 0.5)	<0.001^†^
***Sociodemographic characteristics***							
**Sex**							0.68
Male	470	89.2 (85.4, 92.1)	55	90.8 (78.5, 96.4)	415	88.8 (84.5, 92.1)	
Female	69	10.8 (7.9, 14.6)	7	9.2 (3.6, 21.5)*	62	11.2 (7.9, 15.5)	
**Race/Ethnicity**							0.45
Non-Hispanic White	485	85.7 (80.7, 89.6)	57	90.2 (75.3, 96.5)	428	84.6 (79.3, 88.8)	
Non-Hispanic Black	14	4.6 (2.4, 8.4)*	^	^	13	4.5 (2.4, 8.5)*	
Hispanic	26	7.2 (4.7, 11.0)	3	4.0 (0.9, 16.2)*	23	8.0 (5.0, 12.5)	
Non-Hispanic other^a^	14	2.5 (1.3, 4.7)*	^	^	13	2.8 (1.4, 5.5)*	
**Region**							0.07
Northeast	56	14.6 (11.2, 18.9)	5	13.6 (4.3, 35.5)*	51	14.9 (11.2, 19.4)	
Midwest	137	27.6 (23.4, 32.3)	20	38.5 (24.7, 54.4)	117	25.0 (21.1, 29.4)	
South	128	27.1 (22.2, 32.7)	9	14.5 (6.7, 28.6)*	119	30.2 (24.9, 36.0)	
West	218	30.6 (25.3, 36.5)	28	33.4 (19.6, 50.7)	190	30.0 (25.0, 35.4)	
**Metropolitan area**							0.58
Metropolitan	397	80.0 (74.6, 84.4)	47	73.8 (54.5, 86.8)	350	81.5 (76.8, 85.4)	
Non-metropolitan	139	19.4 (15.0, 24.7)	14	25.0 (12.1, 44.6)*	125	18.1 (14.3, 22.6)	
Not identified	3	0.6 (0.1, 2.9)*	^	^	^	^	
**Education**							0.09
<High school	28	5.7 (3.5, 9.0)	3	3.6 (0.7, 16.3)*	25	6.2 (3.8, 9.9)	
High school degree or GED	157	32.7 (27.3, 38.6)	22	37.8 (21.6, 57.2)	135	31.5 (26.6, 36.8)	
Some college/associate degree	178	32.9 (27.5, 38.8)	25	42.2 (27.0, 59.1)	153	30.7 (25.5, 36.3)	
≥Bachelor's degree	176	28.7 (24.0, 33.9)	12	16.4 (8.1, 30.3)*	164	31.7 (27.0, 36.8)	
***Tobacco use behaviors***							
**Cigarette smokers**							<0.001^†^
Current cigarette smokers	119	21.6 (17.9, 25.8)	12	12.0 (5.4, 24.6)*	107	23.9 (19.7, 28.8)	
Former cigarette smokers	212	34.7 (30.4, 39.2)	13	13.0 (6.5, 24.5)*	199	39.9 (34.5, 45.5)	
Never cigarette smokers	208	43.7 (39.2, 48.4)	37	75.0 (61.3, 85.0)	171	36.2 (31.0, 41.7)	
Indeterminate	0	0.0 (0.0, 0.0)	0	0.0 (0.0, 0.0)	0	0.0 (0.0, 0.0)	
**ENDS users**							0.01^†^
Current ENDS users	89	20.7 (15.6, 26.8)	29	43.9 (26.9, 62.4)	60	15.1 (11.0, 20.3)	
Former ENDS users	190	35.2 (30.2, 40.6)	16	31.7 (18.3, 48.9)	174	36.1 (30.5, 42.0)	
Never ENDS users	260	44.1 (38.8, 49.5)	17	24.5 (13.9, 39.4)	243	48.9 (43.0, 54.7)	
Indeterminate	0	0.0 (0.0, 0.0)	0	0.0 (0.0, 0.0)	0	0.0 (0.0, 0.0)	
**Smokeless tobacco users**							0.63
Current smokeless tobacco users	141	27.5 (22.9, 32.5)	19	25.2 (13.4, 42.3)	122	28.0 (23.2, 33.4)	
Former smokeless tobacco users	227	40.1 (34.3, 46.1)	18	35.4 (21.4, 52.4)	209	41.2 (35.0, 47.7)	
Never smokeless tobacco users	171	32.5 (27.3, 38.1)	25	39.4 (24.0, 57.3)	146	30.8 (25.5, 36.6)	
Indeterminate	0	0.0 (0.0, 0.0)	0	0.0 (0.0, 0.0)	0	0.0 (0.0, 0.0)	
**Use in the past 30 days** ^**b**^	501	92.2 (88.8, 94.7)	56	87.9 (74.0, 94.9)	445	93.3 (89.7, 95.7)	0.32
**Most often used brand in past 30 days**^**c**^ (only 1 selection allowed)							
ZYN	241	46.9 (40.7, 53.2)	35	65.3 (49.0, 78.7)	206	42.7 (37.0, 48.7)	0.01^†^
on!	99	19.7 (15.7, 24.4)	8	13.6 (5.7, 29.2)*	91	21.0 (16.8, 26.1)	0.24
Velo	19	5.1 (3.0, 8.5)	^	^	18	4.9 (3.0, 8.1)	0.86
Rogue	48	8.8 (6.3, 12.1)	5	4.0 (1.3, 11.7)*	43	9.8 (6.8, 14.0)	0.07
Other	80	16.5 (12.5, 21.4)	5	8.6 (3.1, 21.9)*	75	18.2 (14.0, 23.4)	0.04^†^
Missing/unknown	14	3.1 (1.8, 5.3)	^	^	12	3.2 (1.7, 5.9)*	0.78
**Flavor(s) used in past 30 days**^**c**^ (can select >1 flavors)							
Menthol	33	7.7 (5.2, 11.4)	3	5.0 (1.0, 21.0)*	30	8.4 (5.7, 12.1)	0.41
Mint	250	49.3 (43.3, 55.3)	34	68.9 (51.8, 82.1)	216	44.8 (38.6, 51.2)	0.02^†^
Clove, spice, or herb	25	3.9 (2.1, 7.1)*	3	4.3 (1.1, 15.2)*	22	3.8 (2.0, 7.0)*	0.86
Fruit	46	6.8 (4.6, 9.9)	3	3.2 (1.0, 10.0)*	43	7.6 (5.0, 11.3)	0.10
Unflavored	69	18.3 (14.0, 23.5)	6	16.1 (6.4, 34.9)*	63	18.8 (14.1, 24.6)	0.72
Top coded	119	22.4 (17.5, 28.3)	9	15.9 (5.6, 37.7)*	110	23.9 (18.8, 29.8)	0.31

Note: unweighted number and weighted % (95% CI) presented.

The sample includes all self-respondent current nicotine pouch users with no exclusions made for indeterminate responses, including no response, don’t know, and refused.

**Every day plus some day users. Current some day or every day use is determined among respondents by the question “Do you NOW use nicotine pouches every day, some days or not at all?”

*Relative standard error (RSE) ≥30%.

^Cells with a sample size of 1 or 2 are suppressed.

^1^P-value for comparison between young adult and adult current users of nicotine pouches.

†Indicates statistical significance (p < 0.05) from the χ2 test.

^a^Non-Hispanic American Indian and Alaskan Native, Asian, Hawaiian or Pacific Islander, and persons of multiple races are combined into one category due to sample size and analytic concerns.

^b^Past 30-day use is determined among respondents by the question “On how many of the past 30 days did you use nicotine pouches?” If a respondent used nicotine pouches on ≥ 1 of the past 30 days, they were characterized as having nicotine pouch use in the past 30 days.

^c^Data on brand and flavors used were collected among every day users or some day users who used in past 30 days.

Due to a low response rate, Rush, NIIN, FRĒ, and Bidi Pouches were top coded.

Due to a low response rate, alcoholic drinks; candy, dessert, or other sweets flavor; tobacco flavor; and some other type of flavor were top coded and are collectively represented in the top coded category.

When stratified by other tobacco product use, current nicotine pouch use was highest among people who currently use smokeless tobacco (10.5% [95% CI: 8.5%, 12.9%]), followed by people who formerly used smokeless tobacco (3.6% [95% CI: 3.0%, 4.4%]), people who use any tobacco products (1.2% [95% CI: 1.1%, 1.3%]), and people who currently smoke cigarettes (1.1% [95% CI: 0.9%, 1.3%]). Nicotine pouch use was rare (0.0% [95% CI: 0.0, 0.1%], unweighted n = 20) among individuals who never used another tobacco product (Table 3).

**Table 3:** Current nicotine pouch use** by use status of cigarettes, smokeless tobacco, never and ever use of tobacco products, 2022-2023 TUS-CPS.

	Current nicotine pouch users**																
	Current cigarette smokers (n=11,035)		Former cigarette smokers (n=23,799)		Never cigarette smokers (n=78,878)		P-value^1^	P-value^2^	Current smokeless tobacco users (n=1,573)		Former smokeless tobacco users^¶^ (n=6,400)		P-value^3^	Never users of any tobacco products (besides nicotine pouches) (n=66,142)		Users of any tobacco products^§^ (including nicotine pouches) (n=47,009)	
	Unweighted no.	% (95% CI)	Unweighted no.	% (95% CI)	Unweighted no.	% (95% CI)			Unweighted no.	% (95% CI)	Unweighted no.	% (95% CI)		Unweighted no.	% (95% CI)	Unweighted no.	% (95% CI)
**Prevalence of current use in sample**	119	1.1 (0.9, 1.3)	212	0.9 (0.7, 1.1)	208	0.3 (0.2, 0.3)	0.13	<0.001^†^	141	10.5 (8.5, 12.9)	227	3.6 (3.0, 4.4)	<0.001^†^	20	0.0 (0.0, 0.1)	539	1.2 (1.1, 1.3)
***Sociodemographic characteristics***																	
**Sex**							0.01^†^	0.002^†^					0.72				
Male	84	75.1 (63.7, 83.9)	188	90.9 (85.2, 94.5)	198	94.8 (89.1, 97.6)			134	94.9 (89.0, 97.7)	215	95.9 (91.1, 98.1)		20	100.0 (100.0, 100.0)	470	89.2 (85.4, 92.1)
Female	35	24.9 (16.1, 36.3)	24	9.1 (5.5, 14.8)	10	5.2 (2.4, 10.9)^*^			7	5.1 (2.3, 11.0)^*^	12	4.1 (1.9, 8.9)^*^		0	0.0 (0.0, 0.0)	69	10.8 (7.9, 14.6)
**Race/Ethnicity**							0.15	0.10					0.75				
Non-Hispanic White	100	76.9 (65.2, 85.6)	192	88.5 (81.3, 93.2)	193	87.8 (79.1, 93.2)			126	85.4 (74.0, 92.3)	209	89.6 (83.1, 93.7)		18	78.3 (46.6, 93.7)	485	85.7 (80.7, 89.6)
Non-Hispanic Black	7	9.1 (4.1, 19.1)^*^	^	^	5	5.2 (2.0, 13.2)^*^			3	3.7 (1.0, 13.4)^*^	4	3.5 (1.0, 11.2)^*^		^	^	14	4.6 (2.4, 8.4)^*^
Hispanic	8	10.4 (5.0, 20.2)^*^	10	6.6 (3.0, 13.9)^*^	8	6.1 (2.7, 13.5)^*^			7	8.5 (3.5, 19.2)^*^	7	3.9 (1.8, 8.4)^*^		^	^	26	7.2 (4.7, 11.0)
Non-Hispanic other^a^	4	3.6 (1.1, 11.4)^*^	8	3.9 (1.6, 9.1)^*^	^	^			5	2.5 (0.8, 6.9)^*^	7	3.0 (1.3, 7.0)^*^		0	0.0 (0.0, 0.0)	14	2.5 (1.3, 4.7)^*^
**Age group (year)**							0.08	<0.001^†^					0.91				
18-24	12	10.8 (4.9, 22.2)^*^	13	7.3 (3.7, 13.7)^*^	37	33.3 (23.4, 44.9)			19	17.8 (9.2, 31.6)^*^	18	17.1 (9.8, 28.2)		3	31.3 (9.0, 67.7)^*^	62	19.4 (14.5, 25.6)
25-34	20	14.7 (8.4, 24.4)	61	29.8 (22.4, 38.4)	80	34.4 (27.1, 42.4)			43	33.6 (23.6, 45.3)	77	29.5 (22.0, 38.4)		8	35.7 (14.2, 64.9)^*^	161	28.5 (24.0, 33.5)
35-54	55	47.2 (36.0, 58.8)	94	45.4 (36.8, 54.2)	70	24.2 (17.7, 32.0)			57	34.6 (26.1, 44.2)	100	39.3 (30.9, 48.5)		4	13.2 (3.1, 41.8)^*^	219	36.5 (31.9, 41.3)
≥55	32	27.3 (19.0, 37.7)	44	17.5 (12.8, 23.5)	21	8.2 (4.7, 14.0)			22	14.0 (9.2, 20.9)	32	14.0 (9.3, 20.5)		5	19.8 (6.3, 47.6)^*^	97	15.6 (12.7, 19.0)
**Education**							0.004^†^	0.01^†^					0.02^†^				
<High school	15	14.4 (7.6, 25.6)^*^	8	4.6 (1.9, 10.8)^*^	5	2.2 (0.6, 7.7)^*^			9	6.3 (3.1, 12.4)^*^	8	4.4 (1.6, 11.4)^*^		^	^	28	5.7 (3.5, 9.0)
High school degree or GED	47	41.5 (30.0, 53.9)	60	29.2 (22.2, 37.4)	50	31.2 (23.2, 40.4)			44	35.0 (24.8, 46.7)	53	28.1 (20.7, 36.9)		7	50.4 (24.5, 76.1)	157	32.7 (27.3, 38.6)
Some college/associate degree	39	29.5 (20.2, 40.8)	75	33.8 (26.6, 42.0)	64	33.8 (25.2, 43.7)			50	36.6 (25.7, 49.0)	67	27.6 (20.4, 36.1)		9	37.8 (14.9, 67.8)*	178	32.9 (27.5, 38.8)
≥Bachelor's degree	18	14.6 (8.8, 23.3)	69	32.4 (24.9, 40.9)	89	32.8 (24.9, 41.8)			38	22.2 (15.1, 31.3)	99	39.9 (32.3, 48.0)		^	^	176	28.7 (24.0, 33.9)
**Region**							0.07	0.02^†^					0.99				
Northeast	14	13.6 (7.6, 23.1)	23	18.0 (11.8, 26.5)	19	12.5 (7.8, 19.3)			13	15.4 (7.4, 29.4)*	21	14.1 (9.0, 21.6)		^	^	56	14.6 (11.2, 18.9)
Midwest	33	29.7 (21.4, 39.6)	44	19.8 (14.3, 26.8)	60	32.8 (25.5, 41.0)			38	27.5 (18.8, 38.4)	56	26.9 (20.5, 34.5)		4	38.8 (14.2, 70.7)^*^	137	27.6 (23.4, 32.3)
South	33	34.2 (24.2, 45.9)	53	27.3 (19.8, 36.4)	42	23.5 (16.4, 32.4)			36	28.2 (19.0, 39.8)	53	28.2 (20.6, 37.2)		4	24.0 (7.4, 55.4)^*^	128	27.1 (22.2, 32.7)
West	39	22.5 (14.7, 33.0)	92	34.9 (27.6, 43.1)	87	31.2 (23.1, 40.7)			54	28.8 (20.1, 39.5)	97	30.7 (23.2, 39.5)		10	28.1 (11.0, 55.4)^*^	218	30.6 (25.3, 36.5)
**Metropolitan area**							0.66	0.73					0.74				
Metropolitan	88	77.7 (67.2, 85.5)	154	79.6 (72.0, 85.5)	155	81.4 (72.5, 87.9)			100	75.4 (62.6, 84.9)	169	80.9 (73.1, 86.9)		15	68.3 (32.1, 90.8)	397	80.0 (74.6, 84.4)
Non-metropolitan	30	21.7 (13.9, 32.1)	56	19.1 (13.4, 26.4)	53	18.6 (12.1, 27.5)			40	23.7 (14.3, 36.6)	56	18.1 (12.4, 25.8)		5	31.7 (9.2, 67.9)^*^	139	19.4 (15.0, 24.7)
Not identified	^	^	^	^	0	0.0 (0.0, 0.0)			^	^	^	^		0	0.0 (0.0, 0.0)	3	0.6 (0.1, 2.9)^*^
**Use in the past 30 days^b^**	108	87.1 (77.2, 93.1)	197	92.9 (87.0, 96.3)	196	94.2 (88.8, 97.1)	0.16	0.30	128	92.2 (84.9, 96.1)	220	94.6 (88.7, 97.5)	0.48	20	100.0 (100.0, 100.0)	501	92.2 (88.8, 94.7)
**Most often used brand in past 30 days^c^** (only 1 selection allowed)																	
ZYN	38	28.4 (19.3, 39.7)	99	45.8 (36.9, 55.1)	104	56.2 (46.1, 65.7)	0.01^†^	0.001^†^	52	44.8 (33.2, 57.0)	118	51.0 (42.4, 59.6)	0.37	12	46.4 (21.8, 72.9)^*^	241	46.9 (40.7, 53.2)
on!	25	22.7 (13.7, 35.2)	40	24.5 (17.1, 33.8)	34	14.5 (9.6, 21.4)	0.80	0.13	17	13.0 (7.5, 21.6)	52	25.2 (18.3, 33.6)	0.02†	^	^	99	19.7 (15.7, 24.4)
Velo	4	4.6 (1.4, 13.9)^*^	8	3.7 (1.5, 8.8)^*^	7	6.4 (2.8, 13.9)^*^	0.77	0.70	4	4.9 (1.9, 12.3)^*^	13	6.0 (3.1, 11.2)^*^	0.72	^	^	19	5.1 (3.0, 8.5)
Rogue	7	7.6 (3.7, 15.0)^*^	21	10.4 (5.9, 17.7)	20	8.0 (5.0, 12.6)	0.47	0.73	16	10.5 (6.1, 17.6)	25	11.7 (7.3, 18.2)	0.77	^	^	48	8.8 (6.3, 12.1)
Other	27	28.1 (19.1, 39.1)	25	13.1 (8.3, 20.0)	28	13.8 (8.4, 21.8)	0.02^†^	0.05	33	21.9 (15.1, 30.7)	9	4.7 (2.2, 10.0)^*^	<0.001^†^	3	19.2 (6.7, 43.8)^*^	80	16.5 (12.5, 21.4)
Missing/unknown	7	8.7 (4.1, 17.4)^*^	4	2.5 (1.0, 6.1)^*^	3	1.1 (0.3, 3.4)^*^	0.07	0.06	6	4.8 (2.1, 10.8)^*^	3	1.4 (0.4, 4.6)^*^	0.11	0	0.0 (0.0, 0.0)	14	3.1 (1.8, 5.3)
**Flavor(s) used in past 30 days^c^** (can select ≥1 flavors)																	
Menthol	9	8.8 (3.6, 19.9)^*^	12	8.8 (4.6, 16.1)^*^	12	6.4 (3.2, 12.6)^*^	1.00	0.79	6	3.9 (1.5, 9.6)^*^	17	6.9 (3.9, 11.9)	0.24	2	14.7 (4.2, 40.2)^*^	33	7.7 (5.2, 11.4)
Mint	37	31.3 (21.3, 43.6)	107	48.7 (39.6, 57.9)	106	57.9 (48.4, 66.9)	0.02^†^	0.003^†^	63	51.5 (39.6, 63.2)	117	56.3 (47.0, 65.2)	0.52	15	73.7 (47.8, 89.5)	250	49.3 (43.3, 55.3)
Clove, spice, or herb	6	3.4 (1.1, 10.3)^*^	10	5.1 (2.1, 11.9)^*^	9	3.1 (1.3, 7.4)^*^	0.58	0.77	4	3.5 (1.0, 11.0)^*^	13	3.8 (1.7, 8.6)^*^	0.87	0	0.0 (0.0, 0.0)	25	3.9 (2.1, 7.1)^*^
Fruit	8	6.0 (2.3, 14.6)^*^	25	10.1 (5.9, 16.9)	13	4.5 (2.3, 8.9)^*^	0.29	0.23	7	2.5 (0.9, 6.7)^*^	26	9.5 (5.7, 15.6)	0.01^†^	^	^	46	6.8 (4.6, 9.9)
Unflavored	25	31.9 (21.1, 45.0)	14	11.0 (6.2, 18.9)	30	17.7 (10.9, 27.6)	0.003^†^	0.01^†^	17	19.4 (11.1, 31.5)	19	10.7 (5.4, 20.1)^*^	0.19	^	^	69	18.3 (14.0, 23.5)
Top coded	36	25.7 (17.0, 36.8)	48	27.2 (19.7, 36.4)	35	17.1 (10.3, 27.0)	0.81	0.17	35	26.2 (15.9, 40.1)	53	25.5 (18.7, 33.7)	0.91	^	^	119	22.4 (17.5, 28.3)

Note: unweighted number and weighted % (95% CI) presented.

The sample includes all self-respondents with no exclusions made for indeterminate responses, including no response, don’t know, and refused.

**Every day plus some day users. Current some day or every day use is determined among respondents by the question “Do you NOW use nicotine pouches every day, some days or not at all?”

*Relative standard error (RSE) ≥30%.

^Cells with a sample size of 1 or 2 are suppressed.

^1^P-value for comparison between current and former cigarette smokers reporting current use of nicotine pouches.

^2^P-value for comparison between current, former, and never cigarette smokers reporting current use of nicotine pouches.

^3^P-value for comparison between current and former smokeless tobacco users reporting current use of nicotine pouches.

†Indicates statistical significance (p < 0.05) from the χ^2^ test.

¶Determined based on the number of ever smokeless tobacco users subtract number of current smokeless tobacco users.

§This group consist of users of the following tobacco products: cigarettes, large/regular cigars/cigarillos/little filtered cigars, regular pipe/hookah/waterpipe, electronic nicotine delivery systems, smokeless tobacco, nicotine pouches, and heated tobacco products.

^a^Non-Hispanic American Indian and Alaskan Native, Asian, Hawaiian or Pacific Islander, and persons of multiple races are combined into one category due to sample size and analytic concerns.

^b^Past 30-day use is determined among respondents by the question “On how many of the past 30 days did you use nicotine pouches?” If a respondent used nicotine pouches on  ≥ 1 of the past 30 days, they were characterized as having nicotine pouch use in the past 30 days.

^c^Data on brand and flavors used were collected among every day users or some day users who used in past 30 days.

Due to a low response rate, Rush, NIIN, FRĒ, and Bidi Pouches were top coded.

Due to a low response rate, alcoholic drinks; candy, dessert, or other sweets flavor; tobacco flavor; and some other type of flavor were top coded and are collectively represented in the top coded category.

The prevalence of ever use in the overall sample remained similar between September 2022 (2.9% [95% CI: 2.7%, 3.1%]), January 2023 (3.0% [95% CI: 2.7%, 3.2%]), and May 2023 (2.7% [95% CI: 2.5%, 2.9%]). However, there was a significant difference in current use among people who ever use across September 2022 (12.6% [95% CI: 10.5%, 15.0%]), January 2023 (15.4% [12.8%, 18.4%]), and May 2023 (19.7% [95% CI: 16.6%, 23.2%]). ZYN remained the most often brand used across survey administration months among people who used nicotine pouches in the P30D (September 2022: 41.7% [95% CI: 32.9%, 51.1%], January 2023: 52.6% [95% CI: 42.6%, 62.3%], May 2023: 45.7% [95% CI: 35.6%, 56.2%]). Among those who reported nicotine pouch use in the P30D, use of on! was higher in September 2022 (23.4% [95% CI: 15.8%, 33.2%]) than in January 2023 (18.4% [95% CI: 12.3%, 26.6%]), and similar in May 2023 (18.2% [95% CI: 12.1%, 26.5%]). Additionally, among people who use nicotine pouches in the P30D, use of fruit flavored nicotine pouches was higher in each successive survey administration month. In September 2022, 4.6% (95% CI: 2.4%, 8.8%) of people who use nicotine pouches in the P30D reported fruit flavor use, compared to 6.1% (95% CI: 3.0%, 12.1%) in January 2023, and 8.7% (95% CI: 5.1%, 14.6%) in May 2023. While not significant, the clove, spice, and herb flavor category use was 1.4% [95% CI: 0.4%, 4.3%] in September 2022, 5.1% [95% CI: 2.2%, 11.4%] in January 2023, and 4.5% [95% CI: 2.0%, 9.8%] in May 2023. The use of unflavored nicotine pouches was higher across the three survey administration months [September 2022: (15.9% [95% CI: 9.4%, 25.7%]), January 2023: (17.7% [95% CI: 10.3%, 28.6%]), May 2023: (20.3% [95% CI: 14.1%, 28.4%])] (Table 4).

**Table 4:** Nicotine pouch use by ever, current some day and every day, and past 30-day use by survey administration month, 2022-2023 TUS-CPS, (N = 114,206).

		September 2022 (n=39,558)		January 2023 (n=37,580)		May 2023 (n=37,068)		P-value^1^
		Unweighted no.	% (95% CI)	Unweighted no.	% (95% CI)	Unweighted no.	% (95% CI)	
**Ever use**	**Prevalence of use^a^ in overall sample**	1,246	2.9 (2.7, 3.1)	1,177	3.0 (2.7, 3.2)	1,119	2.7 (2.5, 2.9)	0.17
	***Sociodemographic characteristics***							
	**Sex**							0.10
	Male	917	74.9 (71.7, 77.8)	889	78.0 (75.3, 80.5)	835	79.5 (76.3, 82.3)	
	Female	329	25.1 (22.2, 28.3)	288	22.0 (19.5, 24.7)	284	20.5 (17.7, 23.7)	
	**Race/Ethnicity**							0.42
	Non-Hispanic White	1,068	82.8 (79.7, 85.5)	1,003	80.7 (77.3, 83.8)	962	82.2 (78.9, 85.1)	
	Non-Hispanic Black	56	5.0 (3.8, 6.6)	38	4.7 (3.2, 6.7)	45	5.7 (4.0, 8.1)	
	Hispanic	60	6.4 (4.8, 8.6)	75	8.7 (6.7, 11.4)	67	7.9 (6.2, 10.1)	
	Non-Hispanic other^b^	62	5.7 (4.1, 7.9)	61	5.9 (4.3, 8.0)	45	4.1 (3.0, 5.6)	
	**Age group (year)**							0.11
	18-24	74	10.3 (8.2, 13.0)	72	12.1 (9.7, 15.1)	76	10.3 (7.8, 13.6)	
	25-34	288	24.8 (21.8, 28.1)	255	21.4 (18.5, 24.5)	246	24.1 (21.1, 27.4)	
	35-54	487	38.2 (35.2, 41.3)	469	40.4 (36.9, 44.0)	399	34.9 (31.7, 38.3)	
	≥55	397	26.6 (23.8, 29.7)	381	26.1 (23.4, 29.0)	398	30.6 (27.5, 33.9)	
	**Education**							0.24
	<High school	64	4.9 (3.7, 6.5)	63	5.1 (3.8, 6.9)	82	6.3 (4.8, 8.4)	
	High school degree or GED	336	30.0 (26.3, 33.9)	362	34.0 (30.8, 37.4)	310	33.2 (29.7, 36.8)	
	Some college/associate degree	449	34.5 (30.9, 38.2)	420	34.2 (30.9, 37.6)	385	33.1 (30.0, 36.4)	
	≥Bachelor's degree	397	30.7 (27.9, 33.6)	332	26.7 (23.7, 29.9)	342	27.4 (24.3, 30.8)	
	**Region**							0.81
	Northeast	138	12.3 (10.2, 14.8)	141	13.8 (11.3, 16.6)	132	14.5 (12.0, 17.5)	
	Midwest	299	25.6 (22.1, 29.3)	306	26.8 (24.2, 29.6)	252	24.5 (21.6, 27.6)	
	South	395	35.7 (31.8, 39.7)	346	32.7 (29.1, 36.4)	375	34.6 (31.1, 38.3)	
	West	414	26.5 (23.6, 29.6)	384	26.7 (23.8, 29.9)	360	26.4 (23.2, 29.9)	
	**Metropolitan area**							0.11
	Metropolitan	946	82.8 (79.9, 85.3)	861	80.0 (76.8, 82.9)	822	79.9 (76.7, 82.8)	
	Non-metropolitan	282	15.6 (13.3, 18.4)	304	19.0 (16.0, 22.3)	282	19.2 (16.3, 22.4)	
	Not identified	18	1.6 (0.9, 2.9)*	12	1.0 (0.4, 2.4)*	15	0.9 (0.5, 1.8)*	
**Current use**	**Prevalence of current use in overall sample^c^**	169	0.4 (0.3, 0.4)	181	0.5 (0.4, 0.6)	189	0.5 (0.4, 0.6)	0.04^†^
	**Current use among ever users^c^**	169	12.6 (10.5, 15.0)	181	15.4 (12.8, 18.4)	189	19.7 (16.6, 23.2)	0.003^†^
	**Current some day or every day use^c^**							0.10
	Some day	84	43.9 (34.3, 54.1)	74	34.6 (26.8, 43.3)	98	49.9 (40.5, 59.4)	
	Every day	85	56.1 (45.9, 65.7)	107	65.4 (56.7, 73.2)	91	50.1 (40.6, 59.5)	
**Past 30 day use** **Pase**	**Prevalence of past 30-day use in overall sample^d^**	157	0.3 (0.3, 0.4)	165	0.4 (0.3, 0.5)	179	0.5 (0.4, 0.6)	0.02^†^
	**Most often used brand in past 30 days^e^** (only 1 selection allowed)							
	ZYN	72	41.7 (32.9, 51.1)	83	52.6 (42.6, 62.3)	86	45.7 (35.6, 56.2)	0.30
	on!	34	23.4 (15.8, 33.2)	30	18.4 (12.3, 26.6)	35	18.2 (12.1, 26.5)	0.60
	Velo	7	6.3 (2.7, 14.3)*	4	2.8 (0.8, 8.9)*	8	6.2 (2.5, 14.3)*	0.41
	Rogue	18	11.5 (6.9, 18.6)	13	6.9 (3.5, 13.2)*	17	8.5 (4.7, 15.0)	0.48
	Other	24	15.9 (10.1, 24.1)	26	13.5 (8.3, 21.3)	30	19.2 (12.7, 28.0)	0.52
	Missing/unknown	^	^	9	5.8 (3.0, 11.1)*	3	2.2 (0.7, 6.3)*	0.11
	**Flavor(s) used in past 30 days^e^** (can select >1 flavors)							
	Menthol	13	9.5 (4.7, 18.4)*	9	6.0 (2.7, 12.8)*	11	7.9 (4.2, 14.6)*	0.69
	Mint	85	51.9 (41.7, 61.9)	80	53.2 (43.8, 62.5)	85	44.4 (35.5, 53.7)	0.30
	Clove, spice, or herb	7	1.4 (0.4, 4.3)*	9	5.1 (2.2, 11.4)*	9	4.5 (2.0, 9.8)*	0.20
	Fruit	13	4.6 (2.4, 8.8)*	13	6.1 (3.0, 12.1)*	20	8.7 (5.1, 14.6)	0.31
	Unflavored	20	15.9 (9.4, 25.7)	24	17.7 (10.3, 28.6)	25	20.3 (14.1, 28.4)	0.69
	Top coded	40	25.7 (18.2, 35.1)	40	21.5 (14.7, 30.3)	39	21.0 (13.5, 31.1)	0.64

Note: unweighted number and weighted % (95% CI) presented.

The sample (N = 114,206) includes all self-respondents with no exclusions made for indeterminate responses, including no response, don’t know, and refused.

*Relative standard error ≥30%.

^Cells with a sample size of 1 or 2 are suppressed.

^1^P-value for comparison between survey administration months.

†Indicates statistical significance (p < 0.05) from the χ2 test.

^a^Ever use is determined among respondents by the question “Have you EVER used a nicotine pouch EVEN ONE TIME?”

^b^Non-Hispanic American Indian and Alaskan Native, Asian, Hawaiian or Pacific Islander, and persons of multiple races are combined into one category due to sample size and analytic concerns.

^c^Current some day or every day use is determined among respondents by the question “Do you NOW use nicotine pouches every day, some days or not at all?”

^d^Past 30-day use is determined among respondents by the question “On how many of the past 30 days did you use nicotine pouches?” If a respondent used nicotine pouches on ≥ 1 of the past 30 days, they were characterized as having nicotine pouch use in the past 30 days.

^e^Data on brand and flavors used were collected among every day users or some day users who reported use in past 30 days.

Due to a low response rate, Rush, NIIN, FRĒ, and Bidi Pouches were top coded.

Due to a low response rate, alcoholic drinks; candy, dessert, or other sweets flavor; tobacco flavor; and some other type of flavor were top coded and are collectively represented in the top coded category.

## Discussion

This study provides nationally representative estimates of nicotine pouch use among U.S. adults. In 2022–2023, approximately 3% of U.S. adults (aged 18+) reported ever use of nicotine pouches, and 0.4% reported current use. This is consistent with previous reports [9–11,15]. Across the three cross-sectional months of TUS-CPS survey administration, the proportion of people who ever use who reported current use was 12.6% in September 2022 and 19.7% in May 2023, a significant difference. Sales of nicotine pouches increased over six-fold from approximately 126-million units sold between August and December 2019 to approximately 808-million units sold between January and March 2022 [3]. Despite increased sales, results of this analysis and several additional published manuscripts among youth, young adults, and adults have found that, overall, nicotine pouch use remains low [4,9,11] and people who use nicotine pouches tend to use other tobacco products [16].

Both ever and current nicotine pouch use was higher among those who were male, non-Hispanic White, and aged 35–54. Similarly, among adolescents and young adults aged 15–24, Patel and colleagues found that people who currently use nicotine pouches were more often over 21 years of age, male, and non-Hispanic White [8]. This study found that ever nicotine pouch use was lowest among those aged 18–24, suggesting low young adult use. It is important to monitor and prevent adolescent and young adult use of these products, as early exposure to nicotine can lead to addiction and harm brain development until age 25 [17]. While people who use nicotine pouches were more often male, this study found a lower proportion of males among people who ever use nicotine pouches compared to people who currently use nicotine pouches. This suggests men may be more likely to continue use of nicotine pouches after experimentation.

Among current people reporting P30D nicotine pouch use, the most common brand used was ZYN. When stratified by age, brand of nicotine pouch used varied among young adults (aged 18–24) and adults (aged ≥25). Young adults had a higher proportion of ZYN use (65.3%) compared to adults (aged ≥25) (42.7%), while adults (aged ≥25) had a higher proportion of on! (21.0%) and “other” brand use (18.2%) compared to young adults (aged 18–24) (on!: 13.6%; other: 8.6%). Published literature consistently show ZYN as the most frequently used nicotine pouch brand [18], with Swedish Match comprising the largest percentage of nicotine pouch market share (ZYN nicotine pouches are a subsidiary of Swedish Match North America (SMNA)) [2]. However, our study results point to differences in brand preference by age, highlighting the importance of monitoring the impact of nicotine pouch advertising and brand appeal, particularly among youth and young adults. It is important to monitor the marketing of nicotine pouches, including price promotions and sponsorships by nicotine pouch brands and marketing claims and themes, as increased marketing exposure is associated with increased susceptibility to tobacco use among youth and is correlated with tobacco use behavior [19–21].

Consistent with published literature [18,22], overall, among people who currently report P30D nicotine pouch use, the most used flavor was mint, with 49.3% of people who currently use nicotine pouches reporting mint flavored product use. These findings are consistent with nicotine pouch sales in recent years finding that mint flavors were the most commonly sold flavors across the U.S [2,23]. Mint was the most commonly used nicotine pouch flavor in all survey administration waves; However, fruit and unflavored nicotine pouch use was higher than the previous wave in each successive survey administration wave. Flavor use may contribute to the attractiveness of nicotine pouches among new and already established people who used nicotine pouches. A recently published study using 2022–2023 TUS-CPS data found that use of flavored-only nicotine pouches is common among people who formerly (88.78%), never (84.73%), or occasionally (80.74%) smoked cigarettes [22]. This may suggest that flavored nicotine pouches potentially appeal to people who never smoke; however, published data on flavor use among adult nicotine pouch users is still sparse and the effects of flavored nicotine pouch use on cessation and switching from other tobacco products is unknown.

People who used nicotine pouches tended to use other tobacco products as nicotine pouch use was rarely observed among individuals who had never used another tobacco product. This suggests that adults are not initiating tobacco product use with nicotine pouches, but that nicotine pouches are being used by people who already use tobacco products. Previous literature indicates that people who currently use nicotine pouches also currently use other tobacco products [16]. When compared across patterns of other tobacco product use, current nicotine pouch use was the highest among people who report current and former smokeless tobacco use, with 10.5% of people who currently use smokeless tobacco and 3.6% of people who formerly use smokeless tobacco reporting current nicotine pouch use. Only 1.1% of people who currently smoke cigarettes and 0.9% of people who formerly smoked reported current nicotine pouch use. Published literature has consistently found that people who use smokeless tobacco have an increased likelihood of nicotine pouch use [12,24]. This may be attributed to several reasons. Both nicotine pouches and smokeless tobacco deliver nicotine orally, and are similar in shape and size [25]. Further, people who use smokeless tobacco have an increased likelihood of perceiving nicotine pouches as a potentially less harmful product due to the fact that, unlike smokeless tobacco, nicotine pouches do not contain tobacco leaf [7]. A study examining September 2022 TUS- CPS data found current nicotine pouch use uncommon among adults who report smoking cigarettes, although individuals who currently smoke and attempted to quit in the past year were more likely to report ever and current nicotine pouch use than those who had not tried to quit in the past year [9]. Similarly, a recently published study using the 2022–2023 TUS-CPS data found the highest prevalence of nicotine pouch use among adults who recently quit other tobacco products, with the largest association following smokeless tobacco being among adults who recently quit smoking [11]. These results suggest that adults who smoke cigarettes and/or use smokeless tobacco may be using nicotine pouches for reduction in other tobacco product use or switching to less harmful products. As of January 2026, FDA has authorized the marketing of 26 nicotine pouches [26]. These products have undergone rigorous scientific review and have been found ‘appropriate for the protection of public health’ by FDA [26]. For adults who smoke, switching completely from cigarettes to nicotine pouches may reduce exposure to many harmful chemicals found in cigarettes [26]. Continuous monitoring and surveillance of patterns of nicotine pouch use by age and other tobacco product use, as well as reasons of for use, can help inform FDA regulatory actions. Our current analysis builds on prior observations by assessing differences among age groups in patterns of other tobacco product use. Among adults who currently use nicotine pouches aged ≥25, nearly 40% reported formerly smoking cigarettes, whereas 75.0% of young adults (18–24 years) who currently use nicotine pouches reported never smoking. When factoring ENDS use, approximately 44% of young adults (18–24 years) who use nicotine pouches reported current ENDS use, whereas almost 49% of people who use nicotine pouches aged ≥25 reported never ENDS use. These differences reflect variation in ENDS popularity among young adults versus older adults. Future research will further the understanding of the role of nicotine pouches in smoking cessation and switching behaviors pertaining to nicotine pouch use, and continued surveillance will allow for monitoring of divergence in patterns of and reasons for use across age groups, with particular attention to young people.

This study is subject to several limitations. The data collected was self-reported. Sample size was small for some groups, such as young adults (aged 18–24 years) who currently use nicotine pouches (n = 62), with the subset of P30D young adults who use nicotine pouches (n = 56) being even smaller. Due to sample size restrictions and low prevalence of use, some nicotine pouch brand and flavor data was top coded in TUS-CPS, and therefore not broken out in the results of this study. This descriptive analysis used chi-squared statistical tests to compare user group differences, as noted in the methods. The use of bivariate analysis makes findings potentially susceptible to confounding. Despite these limitations, this study adds to the limited published literature pertaining to nicotine pouches and provides some of the most recent nationally representative estimates of nicotine pouch use in the U.S. While several papers have been published on nicotine pouch use using TUS-CPS data, none provide the level of depth included in this paper. This analysis builds on prior observations of the data through its assessment of differences among age groups in patterns of other tobacco product use and its detailed characteristic breakdown. Further, this study is the first nationally representative study to report on adult nicotine pouch use by brand. The 2022–2023 TUS-CPS data were collected prior to the issuance of Tobacco 21, which became effective in August 2024, raising the federal minimum age for sale of tobacco products, including nicotine pouches, from 18 to 21 years [27].

Despite no difference in prevalence of ever nicotine pouch use in the overall sample, an current nicotine pouch use was higher among ever users in September 2022 than in May 2023. Given these findings and the rapidly evolving tobacco marketplace, particularly pertaining to novel oral nicotine products, ongoing monitoring and surveillance will aid understanding of long-term patterns in nicotine pouch use. Further, published research on nicotine pouch use is limited and largely cross-sectional; little is known about patterns of switching to oral nicotine products among people who currently use tobacco and the impacts of nicotine pouch on dependence. Additional research will help elucidate patterns of nicotine pouch use over time and support effective tobacco regulatory efforts.

## Conclusions

As of 2023, nicotine pouch use was low among U.S. adults and most prevalent among non-Hispanic White males aged ≥25. Nearly all people who use nicotine pouches use other tobacco products. Given the higher proportion of current nicotine pouch use among ever users across successive 2022–2023 survey administration months, ongoing monitoring will aid the understanding of long-term patterns in nicotine pouch use. This study provides the most up-to-date information on patterns of nicotine pouch use among U.S. adults and can inform future surveillance and regulatory efforts regarding nicotine pouches.
